# Strain relaxation index and diastolic function from echocardiography: the multi-ethnic study of atherosclerosis

**DOI:** 10.1186/1532-429X-15-S1-E75

**Published:** 2013-01-30

**Authors:** Bharath Ambale Venkatesh, Anderson C Armstrong, Chia-Ying Liu, Andre Almeida, Eui-Young Choi, Boaz D Rosen, Marcelo Nacif, Colin O Wu, David A Bluemke, Joao A Lima

**Affiliations:** 1 Radiology, Johns Hopkins Hospital, Baltimore, MD, USA; 2 Cardiology, Johns Hopkins Hospital, Baltimore, MD, USA; 3 Radiology and Imaging Sciences, National Institutes of Health, Bethesda, MD, USA; 4 National Institutes of Health, Bethesda, MD, USA

## Background

A novel strain relaxation index (SRI) is introduced to assess diastolic function by CMR, using myocardial deformation during LV relaxation. We investigate how SRI relates to standard diastolic parameters by echocardiography (echo). We also relate SRI to mass to volume ratio (MVR) by CMR, which is seen to increase in diastolic dysfunction. SRI accounts for both very early myocardial relaxation and tissue compliance.

## Methods

**Figure 1:**
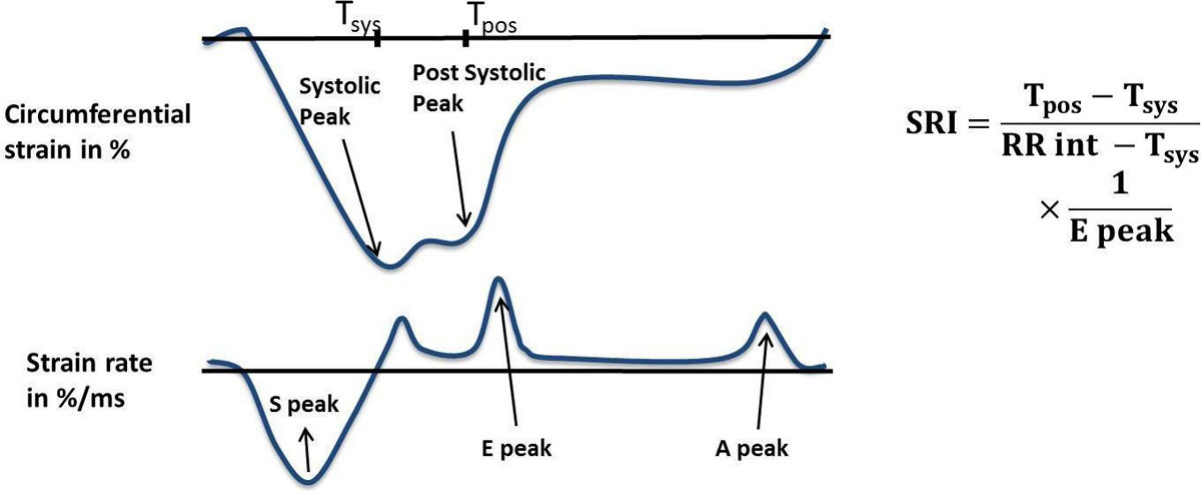
Illustration of the calculation of the proposed SRI from the circumferential strain (top) and strain rate curves (bottom). More negative strain values indicate greater circumferential shortening.

Participants from the Multi-Ethnic Study of Atherosclerosis (MESA) who underwent echo and tagged CMR on the same day at the Johns Hopkins Hospital (2006-2008) were included. Harmonic phase analysis was used to compute mid-ventricular mid-wall circumferential strains and strain rates (Figure 1). SRI was calculated as the difference between post-systolic and systolic times of the strain peaks (indicator of myocardial relaxation), divided by the early diastolic strain rate peak (measure of tissue compliance). It was normalized by the total relaxation time, calculated as the difference between the RR interval and the systolic interval. CMR LV mass and end-diastolic volumes were assessed by the Simpson method. Tissue Doppler echo assessed lateral and septal diastolic tissue velocity (e' wave). For E/e' calculation, echo pulse-wave Doppler E peak was divided by the average of sep and lat e' waves. Diastolic function was rated from 0 to 3, according to the number of matched criteria: (1) septal e' < 8 cm/s; (2) lateral e'< 10cm/s; and (3) E/e' ≥ 10. Pearson's correlation compared SRI to MVR and echo parameters, and ANOVA tested differences across diastolic ratings.

## Results

**Figure 2:**
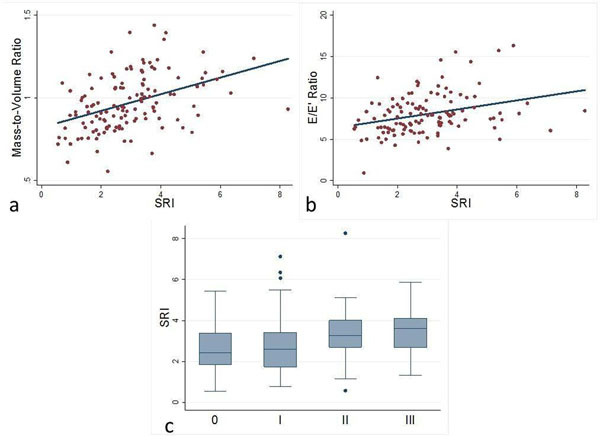
Correlation of SRI and mass-to-volume ratio (a) and E/e' ratio (b). Box plot of SRI measures across grades of diastolic function (c).

We included 125 participants, age 61±8 years; 41% males; 56% White, 44% African-American; 50% hypertensive; 14% diabetic. Mean values for MVR, sep e', lat e', E/e', and SRI were 0.97±0.17 g/mL; 9.1±2.3 cm/s; 10.6±3.1 cm/s; 8.0±2.5; and 2.94±1.4 ms, respectively. SRI correlated positively to MVR (r=0.42, p < 0.001, Figure 2a) and E/e' (r=0.31, p<0.001, Figure 2b), but negatively to e' values (septal r = -0.28, p < 0.001; lateral r = -0.22, p = 0.01). SRI showed increasing trend across diastolic function ratings (p = 0.03, Figure 2c).

## Conclusions

SRI, a novel indicator of diastolic function, as measured from tagged CMR showed good relation to MVR by CMR and standard echo parameters of diastolic function.

## Funding

NHLBI grant - HL066075.

